# Large-volume *en-bloc* staining for electron microscopy-based connectomics

**DOI:** 10.1038/ncomms8923

**Published:** 2015-08-03

**Authors:** Yunfeng Hua, Philip Laserstein, Moritz Helmstaedter

**Affiliations:** 1 Department of Connectomics, Max Planck Institute of Brain Research, Max-von-Laue-Strasse 4, D-60438 Frankfurt, Germany.

## Abstract

Large-scale connectomics requires dense staining of neuronal tissue blocks for electron microscopy (EM). Here we report a large-volume dense *en-bloc* EM staining protocol that overcomes the staining gradients, which so far substantially limited the reconstructable volumes in three-dimensional (3D) EM. Our protocol provides densely reconstructable tissue blocks from mouse neocortex sized at least 1 mm in diameter. By relaxing the constraints on precise topographic sample targeting, it makes the correlated functional and structural analysis of neuronal circuits realistic.

## Abstract (web summary)

Large-scale dense reconstruction of neuronal circuits (or connectomics) requires methods for large-volume dense *en-bloc* electron microscopy (EM) staining. Here the authors develop a protocol for staining tissue blocks from mouse neocortex sized at least 1 mm in diameter, enabling correlated functional and structural circuit analyses.

## Graphical abstract

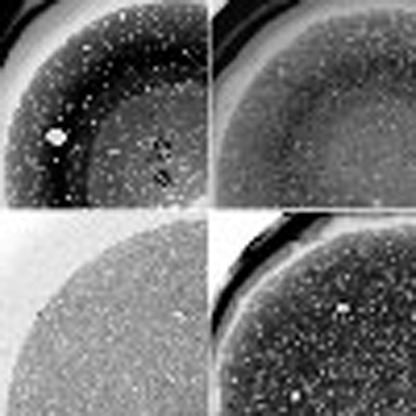

Mapping neuronal circuits densely is the goal of connectomics. Current imaging methods in high-resolution connectomics require *en-bloc* staining of neuronal tissue for large-volume EM^1^. Establishing high-contrast staining in every neurite and synapse in large volumes homogeneously is, however, a substantial challenge to the employed staining protocols. In spite of research into the chemistry of heavy metal deposition in biological samples over several decades, the limitation of the employed protocols to small-dimension samples (typically ∼100–200-μm maximum penetration depth) had been widely accepted. Only very recently, a protocol aimed at whole-brain staining has been proposed^2^, which, however, is focused on very large whole-brain-sized samples and involves staining procedures in the scale of months, not days. We developed a staining protocol that overcomes the limitations of sample size with a simple, fast and reliable process, which provides high-contrast full-neuropil staining for large (millimetre size) volumes.

## Results

**Figure 1:**
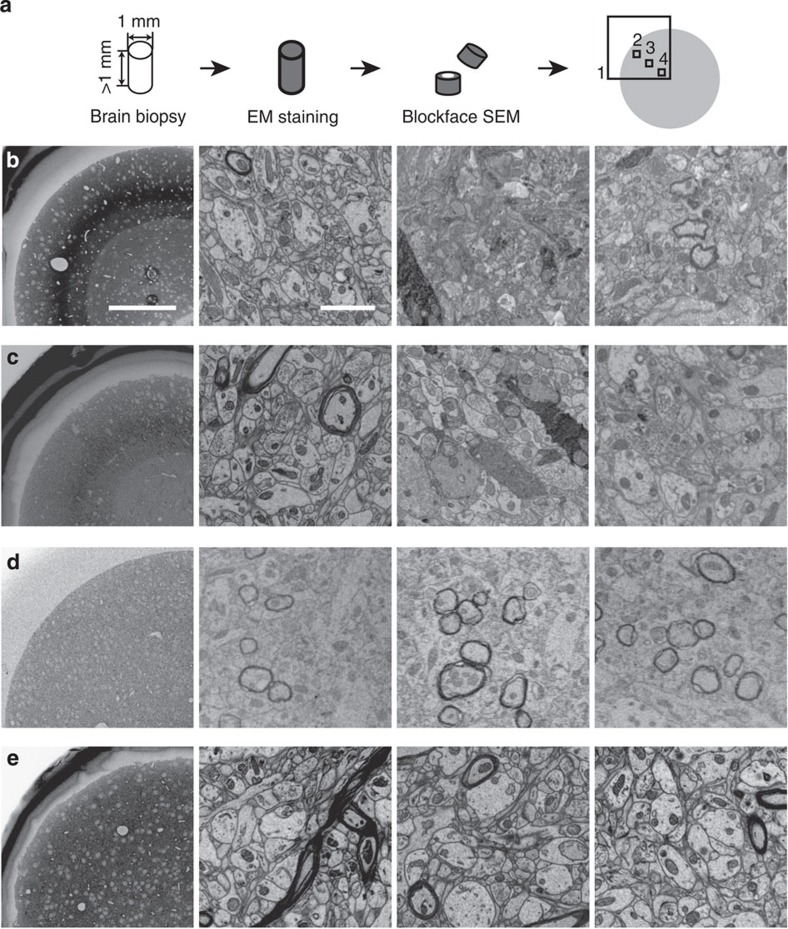
Novel large-scale *en-bloc* EM staining protocol for dense connectomic circuit reconstruction: protocol comparison. (**a**) Screening strategy for brain biopsy samples >1 mm in smallest dimension. Samples were screened after cutting the sample in about half, such that the core of the sample was exposed and could be tested for staining quality. Samples were screened in overview (1), periphery (2), intermediate (3) and core regions (4). (**b**–**e**) Scanning electron microscopy images from mouse cortex biopsy samples screened as indicated in **a** (regions 1–4, left to right columns) and stained with *en-bloc* protocols from ref. 18 (**b**, rOTO), ref. 19 (**c**, rOTO) and ref. 2 (**d**, PATCO) and this protocol (**e**) Note the good staining quality in the periphery (first column) in **b**,**c** but the strong over-staining in the intermediate region yielding stain uptake of a subset of neurons (**b**,**c**) and a substantial staining gradient, which is relieved in this protocol. (**e**) PATCO provides good stain penetration but stains myelinated neurites only, (**d**) Scale bars, 200 μm in **b**–**e** left column and 2 μm in other columns.

The widely employed existing EM staining protocols either are limited to small volumes, yielding prohibitive staining gradients for larger structures (Fig. 1a-c), or stain only a subset of the neurites (myelinated fibre staining, Fig. 1d), with the additional requirement for charging compensation^2^. Our protocol provides, however, high-contrast full-neuropil staining for large (millimetre size) volumes (Fig. 1e). Its chemical logic, key steps and usability for dense circuit reconstruction are described in the following.

Neuronal tissue staining for high-resolution connectomics requires the deposition of heavy metal compounds into the membranes that outline neuronal cell bodies, their dendrites, axons and synaptic vesicles. Osmium (Os) and uranium compounds have been the most widely employed membrane contrasting agents for decades^4^,^5^,^6^, and uranyl acetate (UA) has also been implied in labelling proteins such as those in the postsynaptic density^5^. Alterations to both the Os tetroxide (OsO_4_) and UA impregnation steps are at the core of our protocol.

To achieve high-contrast staining throughout large tissue blocks, the relevant compounds have to diffuse from the exterior through many successive lipid membranes to the centre of the tissue block. Secondly, these compounds have to react with the target structures in the membranes (or be deposited in the membrane by other means). There is a tradeoff between these two goals, since processes that enhance membrane attachment will reduce the availability of compounds for further diffusion, and *vice versa*. This is especially true when enhancing reactions are employed that further increase compound attachment, but thus decrease diffusability. One important enhancing reaction is the application of ferrocyanide for enhanced Os staining, which will be discussed below.

**Figure 2:**
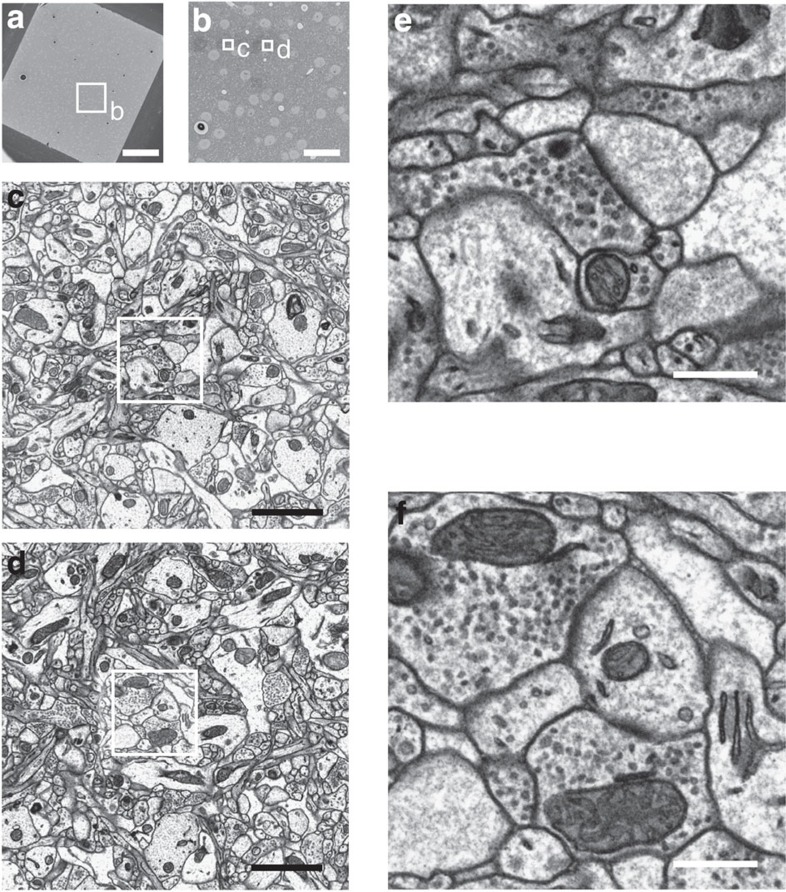
High-resolution screening of ultrastructure and staining contrast at the core of ∼1-mm-sized tissue sample. (**a**–**d**) Position of screening region within sample. (**e**,**f**) Two example high-resolution scanning electron microscopy images (imaged at voxel size of 1.35 × 1.35 nm) showing continuous strong membrane contrast in plasma and vesicle membranes, indications of postsynaptic density, and intracellular organelles. Images in **e**,**f** were imaged first on a freshly cut block face, and images in **a**–**d** were taken afterwards to avoid effects of multiple exposure in the high-resolution images. See Supplementary Fig. 1 for further high-resolution screening results. Scale bars, 200 μm in **a**, 40 μm in **b**, 2 μm in **c**,**d** and 500 nm in **e**,**f**.

In brief, our protocol alterations therefore centre around improving the optimum between tissue penetration and membrane contrast. By separating the OsO_4_ penetration from the ferrocyanide enhancement reaction, by leaving out the intermittent washing step and by using a two-step temperature protocol in the UA impregnation, we achieve our homogeneous high-contrast penetration results as shown in Fig. 1e. Most notably, our protocol completely abolishes the intense staining ring generated in the state-of-the-art *en-bloc* staining protocols (Fig. 1b,c) which had been implied in blocking further stain penetration. When imaged at high resolution (Fig. 2), we find that samples stained with our protocol show intense and continuous membrane contrast (Fig. 2e,f and Supplementary Fig. 1) at about 500 μm from the sample surface (Fig. 2a) with no indication of contrast decrease over this penetration distance.

**Figure 3:**
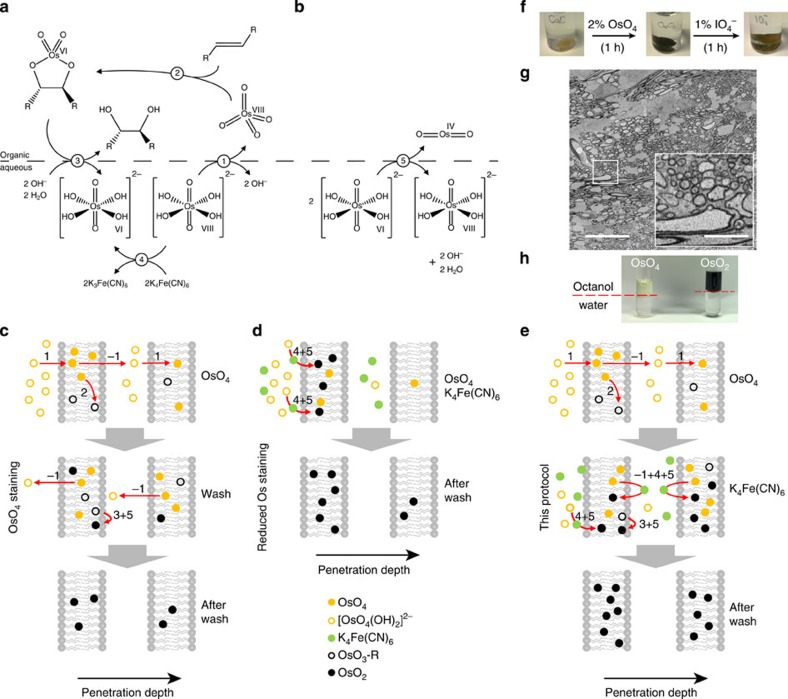
Possible chemical reactions underlying Os-based membrane staining and logic of our staining protocol. (**a**) Sharpless-dihydroxilation (modified from ref. 9) and possible effect of reducing agents such as ferrocyanide. Note that reaction steps 1–5 are referred to in Results, and inverse reaction steps are referred to as ‘−1' to ‘−5'. (**b**) Os^IV^ dismutation suggested to be critical for deposition of OsO_2_ in the membrane (after ref. 12) (**c***–***e**) Possible reaction and diffusion steps involved in stain penetration and contrast enhancement in the conventional OTO protocol, (**c**) rOTO protocol (**d**, see also Fig. 1b,c) and this protocol (**e**, compare with Fig. 1e). Two membranes are visualized to indicate penetration of successive plasma and intracellular membrane bilayers. Only key reaction steps are indicated. (**f**) Simple test experiment that lead us to consider OsO_2_ as a main source of membrane contrast. After Os impregnation, the tissue obtains a dark black colour under bright-field illumination (middle). This is reversed by periodic acid application (right), suggesting that OsO_2_ is oxidized to colourless Os species. (**g**) EM contrast after periodic acid application was decreased, staining only myelinated fibres (inset, note region between myelinated fibres has very low contrast making the detection of single neurites impossible), suggesting that OsO_2_ had been essential for Os membrane contrast. (**h**) Simple octanol/water partition test: equal fractions of OsO_4_ in aqueous solution and octanol partition into organic (top) and aqueous (bottom) phase. After several hours (right) the lipophilic phase is deep black, supporting the notion that OsO_2_ (which is the product of OsO_4_ reduction by octanol) can be dissolved in lipophilic membranes and may contribute crucially to membrane contrast. Scale bars, 20 μm and 5 μm in **g** and insert, respectively.

Our protocol alterations are based on three notions about the underlying diffusion and reaction steps. We report these notions here to illustrate the reasoning that guided our protocol development (Fig. 3). First, we assumed that the classic OsO_4_-alkene reaction mechanism (the Sharpless dihydroxylation reaction^7^,^8^,^9^) is at the core of the OsO_4_–membrane interactions (Fig. 3a). Second, we find indications that in addition to the covalent binding of OsO_4_ to unsaturated lipids, quadruply reduced Os^IV^, made available as OsO_2_ by dismutation of Os^VI^ compounds (Fig. 3b), and dissolved in the membrane, substantially contributes to membrane contrast. Finally, we exploit the fact that the diffusability and reactivity of UA is differentially affected by temperature, such that lower temperature reduces reactivity^10^ more strongly than it reduces diffusibility.

These reaction mechanisms imply that OsO_4_ diffuses across membranes by switching between its ionized hydrophilic form [OsO_4_(OH)_2_]^2−^ and its non-polar lipophilic form OsO_4_ (Fig. 3a, step 1 when entering the membrane, inverted step 1 (‘−1') when exiting the membrane, see sketch in Fig. 3c). After entering the membrane, OsO_4_ can form cyclic osmate esters by covalent binding to unsaturated membrane lipids^11^,^12^,^13^ (Fig. 3a, step 2). However, these intermediates slowly undergo dihydroxylation^11^,^14^ (Fig. 3a, step 3), with the effect of Os leaving the membrane again, yielding [OsO_2_(OH)_4_]^2−^ , a water soluble Os compound at oxidation level VI. This step alone would result in membrane de-staining; however, it has been suggested based on chemical analysis^12^ that instable Os^VI^ compounds dismutate quickly to OsO_4_ and OsO_2_ (Fig. 3b, step 5). OsO_2_ is non-polar and thus likely lipophilic and will be dissolved in the membrane, thus securing in-membrane Os deposition.

Following this logic, the main effect of reducing agents such as potassium ferrocyanide (K_4_Fe(CN)_6_, step 4 in Fig. 3a) is to convert the VIII-oxidized water-soluble Os form into a VI-oxidized water-soluble form^15^, which makes more Os^VI^ available for the dismutation step (Fig. 3b, step 5), and thus generates additional OsO_2_ to be deposited in the membrane. This pathway circumvents the osmate ester formation step (Fig. 3a, step 2), which requires unsaturated membrane lipids as reaction partners^15^.

Together, this would imply that the availability of Os^VIII^ for sequential membrane uptake/loss events (steps 1, −1) is essential for the penetration of membrane stain into the tissue blocks. The generation of reduced Os species (Os^VI^ and Os^IV^) only via the dihydroxilation (Fig. 3a), however, may saturate with decreasing availability of unsaturated membrane lipids. Both effects could explain why the OTO protocol^16^ (which involves two OsO_4_ incubation steps and an intermittent thiocarbohydrazide (TCH) enhancing reaction, but no reducing agent, Fig. 3c) provides good stain penetration but low contrast (limited by availability of unsaturated membrane lipids). In addition, OsO_4_ can be washed out in this protocol during the washing steps following OsO_4_ incubation (via reaction −1).

On the other hand, reduced Os protocols (rOTO^17^; using ferrocyanide/OsO_4_ mixture instead of OsO_4_, also used in recent studies^18^,^19^, Fig. 3d) ‘push' almost the entire water-dissolved Os^VIII^ fraction into the membrane thus avoiding unsaturated lipid depletion and increasing local contrast, but hampering further membrane penetration. This can explain why the reduced Os staining protocols (Fig. 3d) provide very good contrast in the periphery, but with limited penetration (typically <200 μm from the tissue surface).

This logic motivates our alteration of the staining protocol (Fig. 3e): we first allow OsO_4_ to penetrate deep into the tissue (via steps 1, -1) and then immediately apply the reducing agent ferrocyanide (without an intermediate washing step, which would extract Os^VI^ or Os^VIII^ from the membrane).

How did we arrive at this protocol alteration? Initially, we attempted to use periodic acid (which was so far used to stain myelinated fibres^2^,^20^), however, together with OsO_4_, such that periodic acid could attach to dihydroxylated lipids and make the binding site again available for a further round of Os amplification through TCH. However, the effect of periodic acid application after OsO_4_ incubation (Fig. 3f) was opposite: membrane staining contrast got worse (Fig. 3g). We also noted that periodic acid weakened the strong macroscopic tissue staining (black under bright-field illumination, Fig. 3f). Since the most likely source of this heavy black staining is membrane-dissolved or -precipitated OsO_2_ (‘Os black'), we therefore wondered whether in fact periodic acid had oxidized OsO_2_ to water soluble and colourless [OsO_4_(OH)_2_]^2−^.

This made us consider an addition to the classic concept of Os membrane staining. So far, specific chemical binding of OsO_4_ to unsaturated lipids was considered the main source of long-term membrane staining^11^,^12^. But what if membrane-dissolved black-colored OsO_2_ was a parallel and crucial source of membrane contrast? Thus the macroscopically black appearance of the OsO_4_-impregnated brain (Fig. 3f, middle) could result from OsO_2_ enriched in membranes. A simple water/octanol partition test (Fig. 3h) provided support for this hypothesis: when mixing equal parts of water-dissolved 2% OsO_4_ and octanol, the organic and aqueous phases separated (Fig. 3h, left). After several hours, however, the organic phase had turned deep black (Fig. 3h, right). Since 1-octanol can progressively reduce OsO_4_ to OsO_2_, but cannot form stable osmate esters due to the absence of carbon–carbon double bonds the partition of black compounds in the lipophilic phase as seen in the partition test (Fig. 3h) is likely caused by the strongly lipophilic OsO_2_.

In summary, with respect to Os staining, our protocol follows the logic that OsO_4_ needs to stay in VIII-oxidized form for subsequent membrane uptake events and thus tissue penetration; but it needs to be reduced to oxidation level IV (OsO_2_) to be kept in the membrane for high-contrast tissue staining.

With respect to uranium staining, we hypothesized that the temperature dependency of UA–substrate interactions was stronger for the chemical reactions leading to binding of UA to protein and membrane targets^10^ than for its diffusibility through tissue. We therefore exposed the samples to a two-step UA immersion: first overnight at low temperature (4 °C) to allow diffusion over large distances; and then a brief step to 50 °C (for 2 h) to enhance reactivity with protein and membrane targets. The latter enhanced staining contrast (Supplementary Fig. 2). In addition, we optimized the dehydration and resin-embedding steps for larger tissue volumes (see Methods).

The protocol was then controlled for inter-experimentator variability and for variability between cortical areas (four independent experimentators; somatosensory and parietal cortex of mouse, Fig. 1e, Supplementary Fig. 3, Supplementary Table 2).

**Figure 4:**
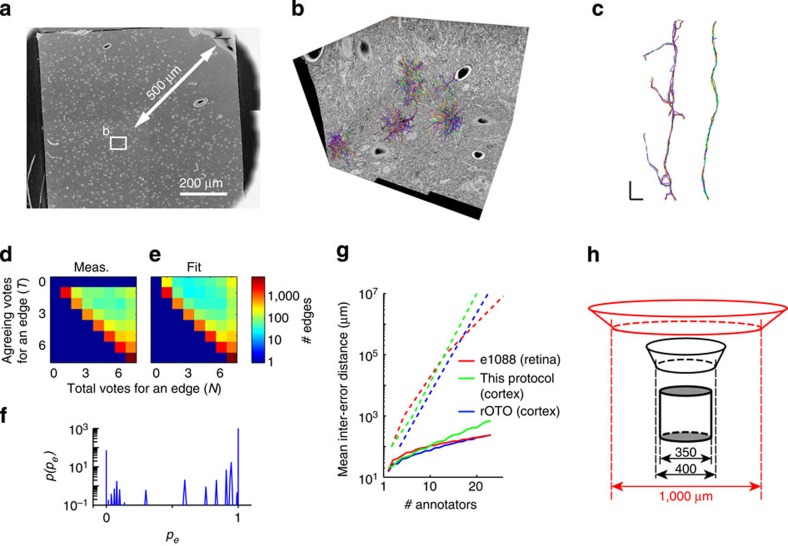
Dense neurite reconstruction in large tissue blocks. (**a**,**b**) Dense neurite reconstruction in a SBEM data set sized 65 × 51 × 41 μm^3^ from the core (>500 μm from the closest sample surface) of a sample from mouse somatosensory cortex. (**a**) Overview of sample location (stitched from four overview images); (**b**) 3D display of SBEM-data set boundaries and all locally dense neurite tracings used for tracing test. (**c**) Two example neurites reconstructed by seven independent annotators. Note complete agreement (right) and local disagreement when tracing spines (left). (**d**,**e**) Measured (**d**) and fitted (**e**) vote histogram of tracer agreement reporting the total number of tracers (*N*) and the agreeing number of tracers (*T*) for each skeleton-edge (redundancy-corrected, see Methods). (**f**) fitted distribution of skeleton-edge difficulty (or edge probability) *p(p*_*e*_) from the tracings summarized in **d**, yielding the fitted vote histogram (**e**, see RESCOP^21^ and Methods for details). (**g**) Prediction of tracing accuracy (for full neurite tracings, continuous lines and for focused re-annotation of the disagreeing locations, dashed lines, see ref. 21) from test tracings for this data set compared with published retina tracings (‘e1088' from ref. 21) and a comparison cortex data set stained with the protocol as shown in Fig. 1b (Boergens *et al*., unpublished data set). (**h**) Illustration of sampling challenge when targeting modules in cortex (here: ‘barrels' in mouse S1 layer 4, grey cylinder) using a protocol that can only stain about 400-μm-wide samples (black) compared with the procedural relief when obtaining about 1-mm-wide samples (red). Scale bars, 200 μm (**a**), 1 μm (**c**).

We finally tested whether the new protocol in fact provides sufficient staining contrast for dense circuit reconstruction (Fig. 4). For this, we acquired a three-dimensional (3D) serial block-face electron microscopy (SBEM) image data set sized 65 × 51 × 41 μm^3^ with a voxel size of 12 × 12 × 30 nm^3^ (Fig. 4b, Supplementary Movie) from the core of one 1-mm-sized sample (at about 500 μm from the sample border, Fig. 4a) and asked 7 trained annotators to start at 116 random neurite seed points in the data set (see Methods for details of the sampling procedure). From each seed point, the annotators were asked to reconstruct the respective neurite within a cubic bounding box of 10 μm edge length (Fig. 4b,c; average traced path length per seed was 27.9 μm including branches), yielding a total tracing of 22.7 mm path length. We then applied the RESCOP skeleton consensus computation^21^, which measures the distribution of agreement between tracers (vote histogram, Fig. 4d). We then used RESCOP to determine the traceability prior (Fig. 4f), which reports the distribution of tracing difficulty in the data set, by fitting to the measured vote histogram (Fig. 4d reports the measured and Fig. 4e the fitted vote histogram). Finally, RESCOP provides a prediction of tracing accuracy in dependence of the number of redundant annotations (Fig. 4g). This quantification allows the comparison to tracing precision in both the published retina data sets^18^,^21^,^22^ and to a data set acquired from mouse somatosensory cortex but stained with the conventional *en-bloc* protocol (protocol from ref. 18, see Fig. 1b; data set: Boergens *et al*., unpublished data set). We find that the dense reconstructions performed at the core of a sample stained with our new protocol are at least as good as those from the retina data set and a conventionally-stained cortex data set (Fig. 4g). Thus, our staining quality is comparable to the previous (volume limited) protocols and allows for dense neuronal circuit reconstruction.

## Discussion

The reported EM staining protocol is currently mainly aimed at yielding consistently high-contrast staining for samples sized about a millimetre in diameter. This is an enormous alleviation of the experimental burden in EM-based connectomics. In many experimental settings, the neuronal tissue-of-interest is a structure of several hundred micrometres in diameter (for example, barrels in mouse S1 cortex, subnuclei in the thalamus or directional preference columns in visual cortex). With the existing protocols, the maximum sample size was just on that same spatial scale, that is, one had to extract samples of about 400 μm in diameter at about 10–50 μm targeting precision from entire mouse brains (Fig. 4h). This represented a substantial obstacle in *en-bloc* EM imaging. Our protocol enables sample volumes that considerably relieve the precision requirements in sample targeting (Fig. 4h).

While such obstacles may be just acceptable for purely structural investigations (where multiple samples can be readily obtained at low resource consumption), this sampling statistics was close to prohibitive for correlated functional/structural experiments. In these settings, the functional experiment alone has usually challenging success statistics (often one-fifth successful experiments). Increasing this sampling burden by a factor of about 5–10 makes such correlated experiments close to impossible. Therefore, our protocol not only makes large-scale structural imaging possible, but substantially alleviates the EM *en-bloc* staining challenges encountered in correlated experimental settings, opening the path to many promising structure-function studies in the emerging field of connectomics.

We tested our protocol for samples sized about 1 mm in diameter. In principle, an extension of the protocol to samples at the size of a hemisphere or an entire mouse brain should be possible by prolonging the respective impregnation steps. With larger samples, however, macroscopic sample damage becomes more likely, such as cracks in the tissue blocks. With high-contrast staining, another challenge is maintaining conductivity throughout large sample volumes, since the less metal deposition in the cytosol (less ‘background') the less conductive the sample. Using conductive resins or increasing cytosol staining may relieve these challenges for very large samples.

Astonishingly, our protocol abolishes a decade-long obstacle to EM staining with only a few targeted modifications of *en-bloc* staining. These were, however, based on particular models of the deposition of Os in biological membranes. At the core is the notion that OsO_2_ substantially contributes to membrane contrast. This is far from proven, and further experiments may elucidate the contributions of Os at low oxidation states to membrane contrast in biological samples. While only tested for neuropil, our protocol may be well-applicable to other forms of tissue, where 3D EM reconstruction is desirable.

## Methods

### Animals

Male mice (P28-P30) were anesthetized and killed by transcardial perfusion of fixative. All procedures were approved by the local animal care and use committee and were in accordance with the laws of animal experimentation issued by the German federal government.

### Sample extraction and fixation

Animals were anesthetized with isoflurane (Baxter) inhalation and perfused with 15 ml cacodylate (Serva, Heidelberg, Germany) buffer (0.15 M, pH 7.4) followed by 30 ml fixative mixture containing 0.08 M cacodylate (pH 7.4), 2.5% paraformaldehyde (Sigma-Aldrich, St Louis, USA), 1.25% glutaraldehyde (Serva) and 2 mM calcium chloride (Sigma-Aldrich). The brain was removed from the skull with care to avoid mechanical irritation and post-fixed for 12 to 24 h at 4 °C in the fixative mixture. Then a biopsy punch (KAI Medical, Honolulu, USA) was used to extract samples sized about 1 mm in diameter and about 2 mm in length.

### Sample staining

Large-volume *en-bloc* staining was performed as follows (also see Supplementary Table 1 for details). Tissue was first immersed in 2% OsO_4_ aqueous solution (Serva) buffered with cacodylate (0.15 M, pH 7.4) at room temperature for 90 min. The staining buffer was then replaced by 2.5% ferrocyanide (Sigma-Aldrich) in 0.15 M cacodylate buffer (pH 7.4) and incubated at room temperature for another 90 min. Sequentially, the tissue was incubated in filtered thiocarbohydrazide (saturated aqueous solution at room temperature, Sigma-Aldrich) at 40 °C for 45 min, 2% unbuffered OsO_4_ aqueous solution at room temperature for 90 min and 1% UA (Serva) aqueous solution at 4 °C overnight. Double rinses in nanopure filtered water for 30 min each were performed between the ferrocyanide and thiocarbohydrazide step, the thiocarbohydrazide and OsO_4_ step, and the OsO_4_ and UA step. On the next day, the tissue (still in UA solution) was warmed up to 50 °C (oven) for 120 min. After being washed twice in nanopure filtered water at room temperature for 30 min, the tissue was incubated in a lead aspartate solution at 50 °C for 120 min. The lead aspartate solution was prepared by dissolving 0.066 g lead nitrate (Sigma-Aldrich) in 10 ml 0.03 M aspartic acid (Serva) and pH adjusted to 5 with 1 N KOH. The tissue was then washed twice in nanopure filtered water for 30 min. The image in Supplementary Fig. 2b was taken from a tissue block stained with the same procedure described above except that the 120 min 50 °C incubation in UA was omitted.

### Sample embedding

For embedding, samples were dehydrated through a graded ethanol series (50, 75, 100%, 30 min each, all cooled at 4 °C) into pure acetone (3 × 100%, 30 min at room temperature) followed by epoxy-monomer infiltration by immersion into 1:1 mixtures of acetone and Spurr's resin (4.1 g ERL 4221, 0.95 g DER 736 and 5.9 g NSA; Sigma-Aldrich) at room temperature for 12 h or overnight on a rotator (samples were maintained in an open Eppendorf tube). Infiltrated samples were then incubated in pure resin with 1% DMAE for 6 h (in closed Eppendorf tubes) and placed in embedding moulds (Polyscience, Eppelheim, Germany) in a pre-warmed oven (70 °C) for 48 to 72 h.

### Sample screening

Embedded samples were trimmed to a block face of 1.5 × 1.5 mm^2^ and imaged in a scanning electron microscope with a field-emission cathode (QuantaFEG 200, FEI Company). An incident electron beam with a spot size of 2.9 and an energy of 2.8 keV was used to scan across the samples at a pixel dwell time of 3.2 μs and a pixel size of 6.1 × 6.1 nm (Fig. 1b,c,e and Supplementary Fig. 2) or 12 × 12 nm (Fig. 1d). Electrons were detected using a custom-designed detector based on a silicon diode (AXUV, International Radiation Detectors) combined with a custom-built current amplifier (courtesy of W. Denk, MPI Heidelberg), as described previously^18^,^22^. To prevent charging for PATCO-stained samples, the chamber was kept at a pressure of 0.20 mbar, while other samples were imaged at high vacuum. All samples were either mounted onto aluminium holders with electrical conductive glue (Henkel, Düsseldorf, Germany) or connected with conductive silver paint (SPI suppliers, West Chester, USA) to the metal stage.

High-resolution images (Fig. 2 and Supplementary Fig. 1) were taken from a tissue block mounted on an aluminium pin with electrically conductive glue. The tissue block was then trimmed at about 500-μm depth from the pia and at about 100 μm from each other side, resulting in a block face with a size of about 970 × 890 μm. The sample was then coated with a gold layer of 200-nm thickness, the block face was exposed again by carefully trimming off the surface with an ultra-microtome (Leica, UC7). EM micrographs were taken in a scanning electron microscope (Verios 460, FEI company) in high vacuum mode. An incident electron beam with an energy of 2.8 keV and a current of 0.2 nA was applied to scan across the sample at a pixel dwell time of 10 μs with a pixel size of 1.35 × 1.35 nm or 5.62 × 5.62 nm.

### SBEM stack acquisition

To test tissue cuttability and for performing tracing test experiments, we acquired a 3D SBEM data set (3 × 3 image tiles; single image size 2,048 × 1,768 pixels; lateral overlap between image tiles about 9% in *x*-direction and 6% in *y*-direction, respectively; 1,370 slices at nominal cutting thickness of 30 nm; incident beam energy 2.8 keV; dwell time 3.2 μs; data set size 65 × 51 × 41 μm^3^; voxel size 12 × 12 × 30 nm^3^) from one of the samples processed with our protocol (Supplementary Table 1, Fig. 4a).

### Neurite reconstruction and traceability computations

To generate tracing seed points, six image regions of size 2 × 2 μm each were randomly selected from the data set. In these regions, all cross-sections of neuronal or glial processes were manually marked. Then, glial processes and cell bodies were excluded, leaving a total of 116 neurite processes. These were delivered to annotators as starting (seed) points. Annotators were asked to trace the respective neurite in all directions starting at the respective seed point, within a cubic bounding box of 10 μm edge length, centred on the seed point. This yielded 116 × 7=812 single skeleton tracings, 22.7 mm total path length. These were then analysed using the redundant skeleton consolidation procedure (RESCOP^21^).

Briefly, each set of seven redundant skeletons was compared computing the number of ‘pro' votes (*T*) and total votes (*N*) for each edge in each skeleton tracing (parameters: ‘initial correspondence test radius' *r*_p_: 225 nm; r.m.s. node distance threshold Θ=375 nm, see code in Supplementary Software). The resulting vote histogram was corrected for the redundancy (*N*) of each tracing, yielding the measured vote histogram (Fig. 4d). Then, the underlying prior of edge probability *p(p*_*e*_) was determined by fitting the vote histogram under the simplifying assumption that tracing decisions were independent between tracers and locations (see ref. 21 for details). The best-fit prior and resulting predicted vote histogram are shown in Fig. 4e,f. Then, the predicted mean error-free path length as a function of the number of tracings per seed point (‘tracing redundancy') was computed from the fitted prior *p(p*_*e*_) (solid lines in Fig. 4g) for the setting of annotators reconstructing an entire neuron/neurite; and for the setting of focused annotation where skeleton edges are repeatedly annotated until a set accuracy goal is reached (dashed lines in Fig. 4g, see ref. 21 for details). The code for this analysis (MATLAB, Mathworks) can be found in the Supplementary Software together with all tracings. For comparison, the published retina data^21^ was re-analysed based on the measured vote histogram using the same code, as was the comparison data set from rOTO-stained mouse cortex (Boergens *et al*., unpublished data set).

### Exploratory test experiments

To ensure that the new protocol is readily applicable to other cortical areas and can be easily implemented by other experimentalists, we asked two students in the laboratory to use the protocol without direct supervision (just based on written instructions). Both were able to conduct successful stainings in somatosensory and parietal cortex (Supplementary Fig. 3 and Supplementary Table 2), though one sample showed charging in parts of the volume (Supplementary Fig. 3c). Successful protocol applications were required between 1 and about 10 training iterations for experienced and novice students, respectively.

## Additional information

**How to cite this article:** Hua, Y. *et al*. Large-volume *en-bloc* staining for electron microscopy-based connectomics. *Nat. Commun*. 6:7923 doi: 10.1038/ncomms8923 (2015).

## Supplementary Material

Supplementary Information Supplementary Figures 1–3 and Supplementary Tables 1–2

Supplementary Software Matlab code and skeleton files for RESCOP analysis of traceability.

Supplementary Movie 1 Movie of 3D SBEM image stacksized 1000×1000×100 voxels (thus about 12 μm x 12 μm × 3 μm) from the mouse cortex test dataset shown in Fig. 4a,b.
