# Supplementary material for: Large-volume en-bloc staining for electron microscopy-based connectomics
Source: Nat Commun. 2015 Aug 3;6:7923. doi: 10.1038/ncomms8923 (PMC4532871; doi:10.1038/ncomms8923)
Supplement: Supplementary Information — Supplementary Figures 1–3 and Supplementary Tables 1–2 [file ncomms8923-s1.pdf]

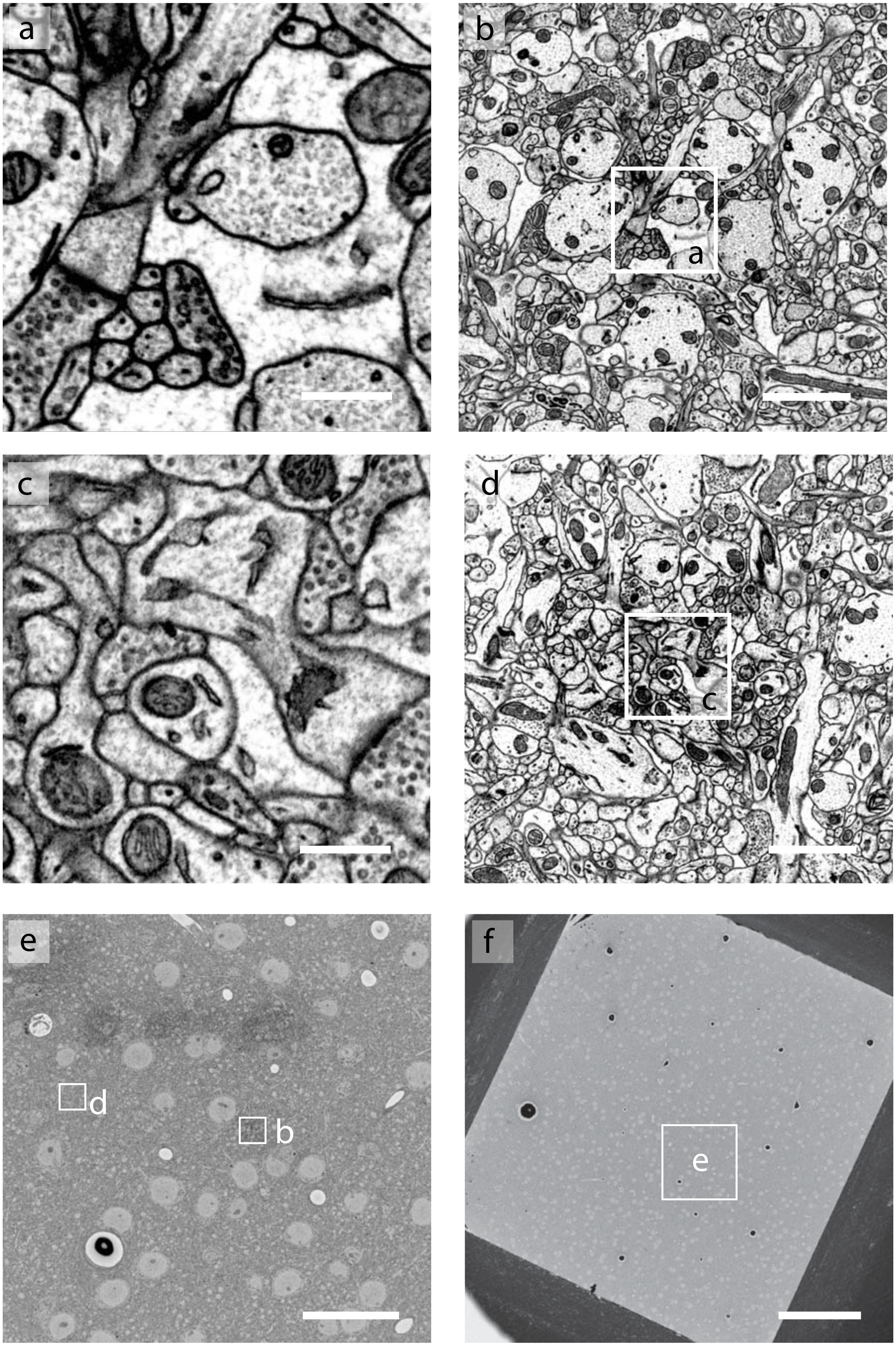

Hua, Laserstein & Helmstaedter
Suppl. Fig. 1

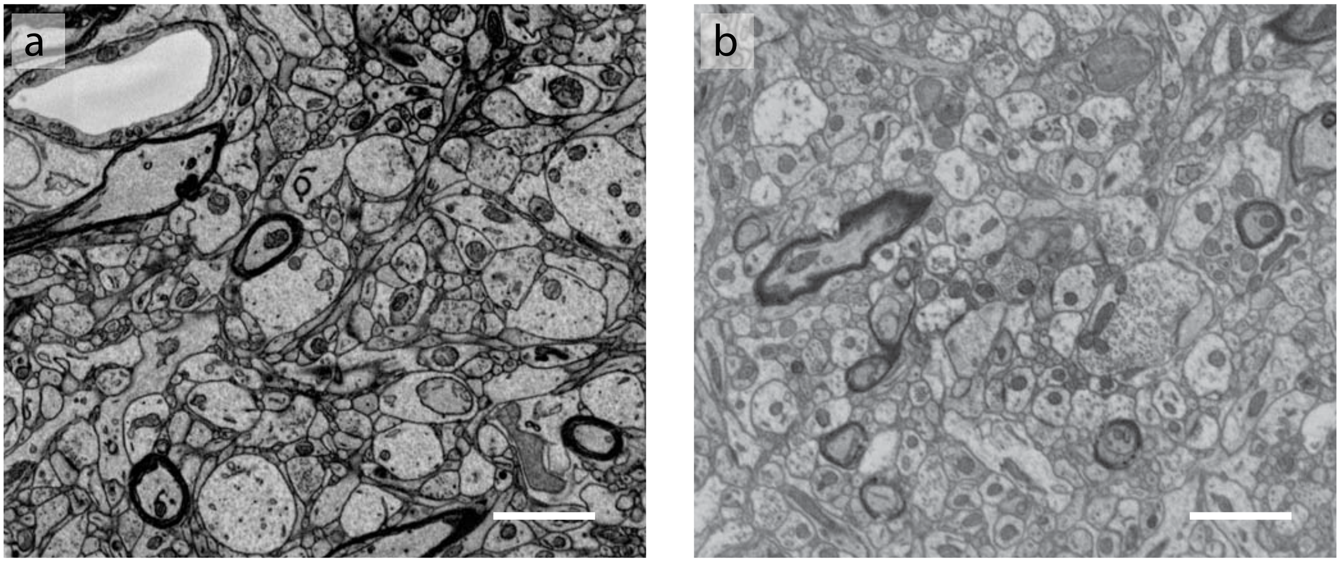

Hua, Laserstein & Helmstaedter
Suppl. Fig. 2

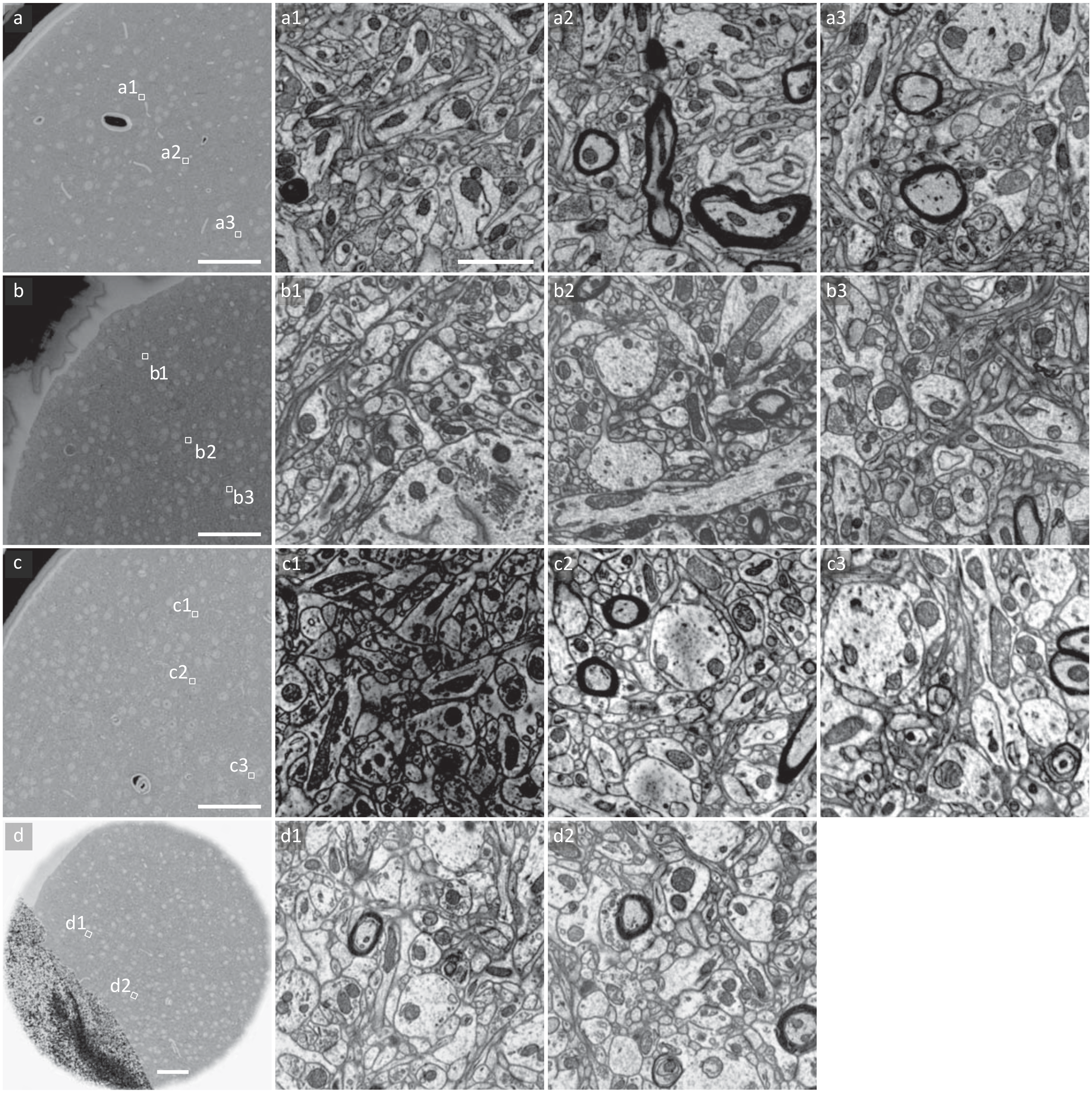

Hua Laserstein Helmstaedter

Suppl. Fig. 3

	staining protocol					
incubation steps	Briggman (Fig. 1b)	Holcomb (Fig. 1c)	Mikula (Fig. 1d)	OTO	OPTO (Fig. 3g)	Hua (Fig. 1e)
1.	2% OsO4,<br>2.5% ferrocyanide,<br>0.15 M Cac, pH 7.4	2% OsO4, 2 mM CaCl2<br>1.5% ferrocyanide,<br>0.15 M Cac, pH 7.4	1 % periodic acide,<br>0.1 M Cac, pH 7.4	2% OsO4,<br>unbuffered	2% OsO4,<br>unbuffered	2% OsO4,<br>0.15 M Cac, pH 7.4
	1.5 h @ rt	1.5 h @ rt	1.5 h @ 4 ºC	1.5 h @ rt	1.5 h @ rt	1.5 h @ rt
	No wash					
2					1 % periodic acide,<br>0.15 M Cac, pH 7.4	2.5% ferrocyanide,<br>0.15 M Cac, pH 7.4
					1.5 h @ 4 ºC	1.5 h @ rt
	0.5 h wash in water x 2					
3	1% TCH,<br>unbuffered	1% TCH,<br>unbuffered	1% TCH,<br>0.1 M Cac, pH 7.4	1% TCH,<br>unbuffered	1% TCH,<br>0.1 M Cac, pH 7.4	1% TCH,<br>unbuffered
	0.75 h @ 50 ºC	0.75 h @ rt	0.75 h @ 50 ºC	0.75 h @ rt	0.75 h @ 50 ºC	0.75 h @ 40 ºC
	0.5 h wash in water x 2					
4	2% OsO4,<br>unbuffered<br>1.5 h @ rt	2% OsO4,<br>unbuffered<br>1.5 h @ rt	2% OsO4,<br>unbuffered<br>1.5 h @ rt	2% OsO4,<br>unbuffered<br>1.5 h @ rt	2% OsO4,<br>unbuffered	2% OsO4,<br>unbuffered
					1.5 h @ rt	1.5 h @ rt
	0.5 h wash in water x 2					
5	1 % uranyl acetate,<br>unbuffered	1% uranyl acetate,<br>unbuffered	1% uranyl acetate,<br>unbuffered	1 % uranyl acetate,<br>unbuffered	1 % uranyl acetate,<br>unbuffered	1% uranyl acetate,<br>unbuffered
	2 h @ 50 ºC	overnight @ 4 ºC	2 h @ 50 ºC	2 h @ 50 ºC	2 h @ 50 ºC	overnight @ 4 ºC,<br>2 h @ 50 ºC
	0.5 h wash in water x 2					
6	Lead aspartate,<br>pH 5.0	Lead aspartate,<br>pH 5.5	Lead aspartate,<br>pH 5.0	Lead aspartate,<br>pH 5.0	Lead aspartate,<br>pH 5.0	Lead aspartate,<br>pH 5.0
	2 h @ 50 ºC	2 h @ 60 ºC	2 h @ 50 ºC	2 h @ 50 ºC	2 h @ 50 ºC	2 h @ 50 ºC
	0.5 h wash in water x 2					
	dehydration, infiltration and embedding					

Supplementary Table 1

Detailed comparison of staining steps in employed staining protocols (Fig. 1).

Experimenter	Species, brain region	Exp. ID	Exp. Date	Variation to standard protocol as in methods	Ultrastructural results	Screening results figure
Hua	Mouse S1 cortex	scYH0009	2013/12/11	rOTO (ref. 18)	Poor penetration, good ultrastructure preservation, good membrane contrast	Fig. 1b
Hua	Mouse S1 cortex	scYH0010	2013/12/11	rOTO (ref. 19)	Poor penetration, good ultrastructure preservation, good membrane contrast	Fig. 1c
Hua	Mouse S1 cortex	scYH0053	2014/01/13	PATCO (ref. 2)	Good penetration, poor ultrastructure preservation, poor membrane contrast, charging	Fig. 1d
Hua	Mouse S1 cortex	scYH0056	2014/01/22	No variation	Penetration, contrast, ultrastructure (all good)<br>resin hardness (35 nm cut at border, soft core)	Fig. 1e<br>Suppl Fig 2a
Hua & Laserstein	Mouse S1 cortex	scPL0052	2014/08/14	No accelerator during initial infiltration, Add accelerator on the next day	Penetration, contrast, ultrastructure (all good)<br>resin hardness (30 nm cut)	Traceability test
Hua	Mouse s1 cortex	scYH1059	2014/01/22	No 2h incubation in UA at 50ºC	Good penetration, good ultrastructure preservation, slightly low membrane contrast	Suppl Fig 2b
Hua	Mouse S1 cortex	scYH1012	2015/05/07	No accelerator during initial infiltration, Added accelerator on the next day instead	Penetration, contrast, ultrastructure (all good)	Fig. 2<br>Suppl Fig. 1
Laserstein	Mouse S1 cortex	scPL0066	2014/08/25	No variation	Penetration, contrast, ultrastructure (all good)<br>resin hardness (not tested)	Suppl Fig. 3a
Gour	Mouse S1 cortex	scAG0008	2014/10/20	No variation	Penetration, contrast, ultrastructure (all good)<br>resin hardness (not tested)	Suppl Fig. 3b
Karimi	Mouse PPC	ppcAK0004	2014/10/21	No variation	Penetration, contrast, ultrastructure (all good), charging effect at the edge area,<br>resin hardness (not tested)	Suppl Fig. 3c
Karimi	Mouse PPC	ppcAK0003	2014/10/21	No variation	Penetration, contrast, ultrastructure (all good)<br>resin hardness (not tested)	Suppl Fig. 3d

Supplementary Table 2

Summary of staining experiments reported in the paper for assessing the variability of staining quality over cortex areas and experimentalists.
